# Intrasubject Gait Variability During Stair Walking in Knee Osteoarthritis: A Kinematic and Kinetic Analysis

**DOI:** 10.1155/abb/6678937

**Published:** 2025-11-26

**Authors:** Ye Ma, Shixin Lin, Yaqi Mao, Chenyi Guo, Dongwei Liu, Meijin Hou

**Affiliations:** ^1^ Research Academy of Grand Health, Faculty of Sports Sciences, Ningbo University, Ningbo, China, nbu.edu.cn; ^2^ Department of Electronic Engineering, Tsinghua University, Beijing, China, tsinghua.edu.cn; ^3^ School of Information Technology and Artificial Intelligence, Zhejiang University of Finance and Economics, Hangzhou, China, zufe.edu.cn; ^4^ National Joint Engineering Research Centre of Rehabilitation Medicine Technology, Fujian University of Traditional Chinese Medicine, Fuzhou, China, fjtcm.edu.cn; ^5^ Key Laboratory of Orthopeadics and Traumatology of Traditional Chinese Medicine and Rehabilitation, Ministry of Education, Fujian University of Traditional Chinese Medicine, Fuzhou, China, fjtcm.edu.cn

**Keywords:** coefficient of variation, kinematics, kinetics, knee osteoarthritis, stair walking

## Abstract

**Background:**

Gait variability in kinematic and kinetic parameters during stair walking is a key indicator of motor function and fall risk in individuals with knee osteoarthritis (KOA). However, normative reference data and pathological patterns in KOA remain under explored.

**Methods:**

This cross‐sectional study analyzed retrospective data from 169 participants, including 116 individuals with KOA and 53 matched healthy controls. Each participant performed both stair ascent and descent tasks, during which lower limb kinematic and kinetic gait parameters were obtained using a three‐dimensional motion capture (3DMC) system and an instrumented staircase. Intrasubject variability, quantified by coefficient of variation (CV), was calculated for all gait parameters. A mixed between–within subject analysis of variance with aligned rank transformed data was conducted to assess the effects of group (KOA vs. control), condition (stair ascent vs. descent), and their interaction.

**Results:**

Individuals with KOA exhibited significantly greater kinematic variability during both stair ascent and descent, whereas greater kinetic variability was observed only in knee and hip joint moments and powers during ascent. Across both groups, variability increased at the knee and distal segments, but decreased at proximal segments (hip and pelvis) during stair ascent compared with descent. KOA individuals displayed distinct adaptation mechanism between stair ascent and descent, but not in kinematic parameters.

**Conclusion:**

Individuals with KOA demonstrate significantly increased movement variability compared with healthy controls during stair walking, especially in knee and hip joint moments and powers during ascent. These findings indicates distinct and task‐specific adaptation strategies in KOA, reflecting altered stability and joint loading mechanisms.

## 1. Introduction

Knee osteoarthritis (KOA) is a highly common degenerative condition that affects various joint structures, including the articular cartilage, synovium, ligaments, subchondral bone, and surrounding muscles [1]. Individuals with KOA typically suffer from pain, joint stiffness, balance dysfunctions, and difficulties in walking [2, 3]. These gait abnormalities have been linked to an increased mortality risk [4]. Mechanical factors play a significant role in KOA progression [5]. Examining gait characteristics in individuals with KOA is crucial for evaluating motor functions, guiding clinical decision‐making, assessing intervention outcomes, and managing degenerative progression. Furthermore, a deeper understanding of dynamic gait features is essential for the design of assistive devices that improve mobility, functionality, and quality of life in this population [5].

Gait analysis is commonly used to examine kinematic or kinetic changes in individuals with KOA during level‐ground walking [6], as these factors are linked to osteoarthritis progression [5]. Compared to healthy individuals, individuals with KOA often exhibit changes in joint kinematics and kinetics, with additional variations observed across different disease severities [7, 8]. Commonly reported kinematic changes in KOA subjects include reduced range of motions at the ankle, knee, and hip joint [6, 9], increased hip, knee, and ankle flexion/extension angles [5], and reduced hip rotation [6]. However, some studies have found no significant differences in knee motion between KOA and healthy groups [10]. In terms of kinetics, KOA patients often display increased knee flexion/extension and adduction/abduction moments [6, 11, 12], as well as increased ankle varus moments [6], alongside reduced hip extension and adduction moments [8, 12]. Inconsistencies also exist in the literature, such as findings of reduced ankle moments and powers [13], and decreased knee moments [8, 13]. Moreover, severe KOA has been associated with reduced knee flexion angle during stance, and decreased knee extension, hip internal rotation, and peak ankle dorsiflexion moments [12].

Gait variability, defined as fluctuations in gait characteristics between steps, reflects the neuromuscular system’s ability, or inability, to maintain a consistent walking pattern [14]. Both increased and deceased variability have been reported in population with gait abnormalities, such as elderly fallers [15, 16], individuals with KOA [17, 18], and patients with neurodegenerative diseases such as Parkinson’s disease [14]. In individuals with KOA, altered gait variability may reflect compensation strategies to reduce joint pain and protect the knee [7]. Such variability has also been associated with impairments in the central nerve system [19], sensory deficits, and balance dysfunction during walking [15, 19, 20]. Accordingly, gait variability has been proposed as a sensitive and clinically relevant measure for evaluating mobility, assessing fall risk, predicting motor disability [19], and monitoring responses to therapeutic interventions [14, 21].

Gait variability in individuals with KOA has been primarily investigated during level‐ground walking, focusing on variability in temporal parameters [22–24], joint angles [23, 25, 26], moments [22], and powers [27] of the lower limb joints and pelvis. However, stair walking, a vital lower limb functional task in activities of daily living (ADL), is often the first to be affected in individuals with KOA [28]. Compared to level‐ground walking, stair walking is more physically demanding, requiring greater movement coordination, joint stability, and muscle strength [28]. This task is therefore particularly challenging for individuals with impaired motor function, such as those with KOA [5, 28]. Therefore, comprehensive biomechanical studies of stair walking in KOA individuals are essential for understanding functional limitations in this population.

To our knowledge, the gait variability in kinematic and kinetic parameters during stair walking in individuals with KOA has not been thoroughly investigated. Therefore, objectives of this study were to: (1) establish normative reference data for intrasubject gait variability in kinematic and kinematic parameters during stair walking (including both stair ascent and descent); (2) investigate pathological gait variability patterns in individuals with KOA compared with healthy controls; (3) evaluate task‐specific adaptive mechanisms in gait variability between stair ascent and descent in both KOA and healthy populations.

## 2. Materials and Methods

### 2.1. Subjects

This cross‐sectional study was based on retrospective data from 169 subjects, including 116 with KOA (age: 58.49 ± 5.97 years; height: 1.60 ± 0.06 m; mass: 59.94 ± 8.10 kg; 14 male and 102 female) and 53 matched healthy controls (age: 59.00 ± 5.97 years; height: 1.62 ± 0.07 m; mass: 61.42 ± 9.36 kg; 21 male and 32 female). KOA was diagnosed as by experienced physicians following the Guidelines for the Diagnosis and Treatment of Osteoarthritis in China [29]. Among the KOA participants, 45 had unilateral involvement and 71 had bilateral involvement, with Kellgren/Lawrence (K/L) grade of two or higher. Exclusion criteria included other lower limb joint pain, lower back pain, rheumatoid arthritis, neuromusculoskeletal disorders, or any other conditions that could affect lower limb function. There is no statistical significant differences in age, height, or body mass between the KOA and control group (*p* = 0.064, 0.14, and 0.25, respectively).

Participants were recruited from local communities in Fuzhou between December 2018 and March 2022. The study protocol was approved by the Ethics Committee of the Affiliated Rehabilitation Hospital, Fujian University of Traditional Chinese Medicine (2018KY‐006‐03 and 2020KS‐5‐1). All subjects were informed about the experiment protocol during recruitment and prior to testing, and written informed consent was obtained from each subject.

### 2.2. Experiments

Standard biomechanical experiments were conducted to record the kinematic and kinetic data during both stair descent and stair ascent in KOA subjects and healthy controls. A customized eight‐step staircase (Figure 1) was designed and manufactured, incorporating two force plates (9260AA, Kistler) embedded in the third and fourth steps to measure ground reaction forces (GRFs) at a sampling frequency of 2000 Hz. The staircase had an inclination of 30.65°, a tread length of 30 cm, and a riser height of 20 cm. The design followed public environment staircase specifications [33], which recommend an inclination between 24° and 42°.

Figure 1Demonstration of the customized staircase and the stair ascent (a) and descent (b) experiment. The staircase was designed at an inclination of 30.65° with the riser height of 20 cm and the tread run of 30 cm. The force plates were embedded in the third and fourth step. The upper limb and lower limb marker set are according to the UWA upper‐body marker set [30, 31] and calibrated anatomical system technique (CAST) protocol [32].

**Figure:**
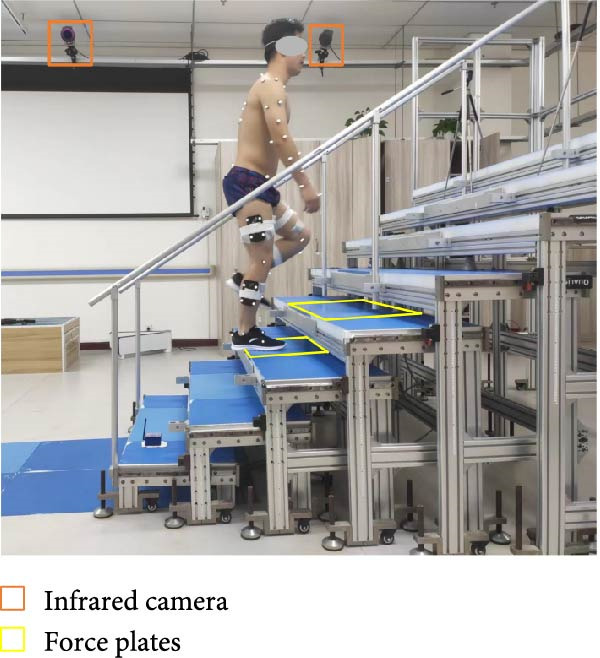
(a)

**Figure:**
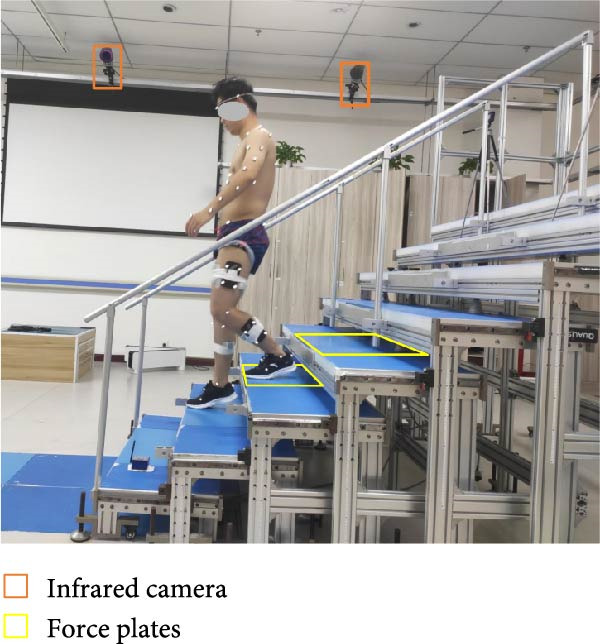
(b)

A three‐dimensional motion capture (3DMC) system consisting of 10 infrared high‐speed cameras (Oqus 700+, Qualisys Track Manager, Sweden) was used to record kinematic data during stair walking at a sampling frequency of 100 Hz. Seventy‐five 14 mm reflective markers were attached on the anatomical landmarks on each subject following the University of Western Australia (UWA) upper‐body marker set and the calibrated anatomical system technique (CAST) protocol [32]. Detailed information on the whole‐body marker configuration is available in [34].

Before data collection, each participant was given several minutes to familiarize themselves with the experimental setup. Each participant then performed a static trial in a predefined anatomical position for at least 6 s to established the inverse kinematic model for each body segment. The static markers were subsequently removed during the dynamic trials. The CAST protocol was employed to correct for the movement artifacts during stair walking [32]. At least eight successful trials were recorded for each subject and for each stair walking condition.

### 2.3. Data Analysis

Kinematic and kinetic parameters were calculated from all trials. Kinematic parameters included foot progression angle and three‐dimensional joint angles of the ankle, knee, hip, and pelvis. Kinetic parameters included GRF in the vertical, anterior–posterior (AP), and medial–lateral (ML) directions, as well as three‐dimensional joint moments and powers of the ankle, knee, and hip joint. GRFs and marker trajectories were first filtered using zero‐lag fourth‐order Butterworth filters with the cut‐off frequencies of 25 and 6 Hz, respectively, as recommended by Winter [35]. Local coordinate systems were established for the head, thorax, trunk, upper arm, forearm, hand, pelvis, thigh, shank, and foot based on the three‐dimensional coordinates of anatomical markers. The foot progression angle and three‐dimensional kinematics were then calculated using an *X*–*Y*–*Z* Euler rotation sequence. Joint moments and powers were determined using standard inverse dynamics procedure [35]. GRFs and joint moments were normalized to body weight (BW), to facilitate comparison between subjects. All kinetic and kinematic calculations were performed using Visual 3D software (C‐Motion Inc., USA).

Stair walking variability was quantified using the coefficient of variation (CV), defined as the standard deviation (Std) of a series of values divided by their absolute mean [36]:

CV=Std vMean v×100%.

For both stair descent and ascent, CVs of all selected kinematic and kinetic parameters were calculated and averaged across the entire gait cycle. The intrasubject CVs, that is, stride‐to‐stride CVs [26], were calculated.

### 2.4. Statistical Analysis

The Kolomogorov–Smirnov test was first conducted to assess the normality of all parameters. Descriptive statistics were presented as mean (Std) for normally distributed parameters, and as median [interquartile range (IQR)] for nonnormally distributed parameters. Since most parameters did not meet the assumption of normality, the main effects of group (KOA vs. normal control group) and condition (stair descent vs. stair ascent) as well as their interaction, were investigated using the analysis of variance of aligned rank transformed data method [37]. The Holm–Bonferroni method was applied to adjust for multiple comparisons. All statistical analyses were performed in R 4.4.0, with the level of significance set at 0.05.

## 3. Results

**Figure 2:**
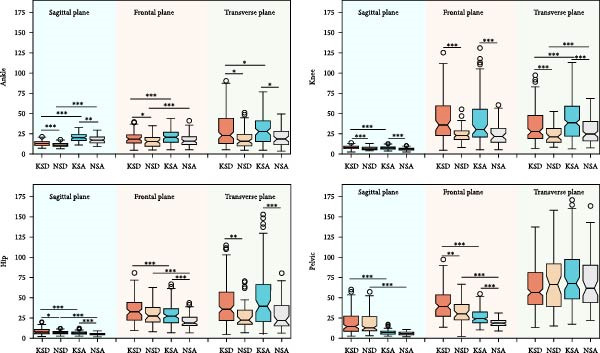
The intrasubject coefficient of variance of lower limb joint angles in sagittal, frontal, and transverse plane. KSD, knee osteoarthritis (KOA) subjects during stair descent (SD); NSD, normal control subjects during SD; KSA, KOA subjects during stair ascent (SA); NSA, normal control subjects during SA. ∗, ∗∗, and ∗∗∗ stand for *p* < 0.05, *p* < 0.01, and *p* < 0.001, respectively.

**Figure 3:**
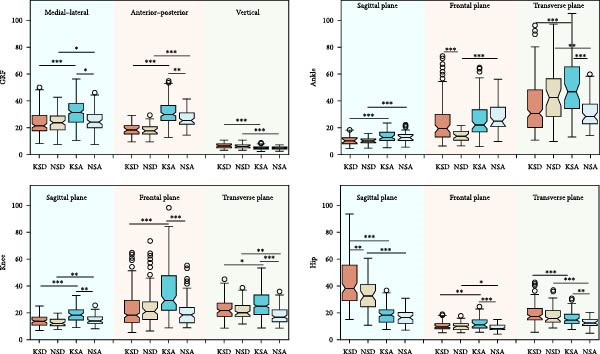
The intrasubject coefficient of variance of three‐dimensional ground reaction forces and lower limb joint moments in sagittal, frontal, and transverse plane. KSD, knee osteoarthritis (KOA) subjects during stair descent (SD); NSD, normal control subjects during SD; KSA, KOA subjects during stair ascent (SA); NSA, normal control subjects during SA. ∗, ∗∗, and ∗∗∗ stand for *p* < 0.05, *p* < 0.01, and *p* < 0.001, respectively.

**Figure 4:**
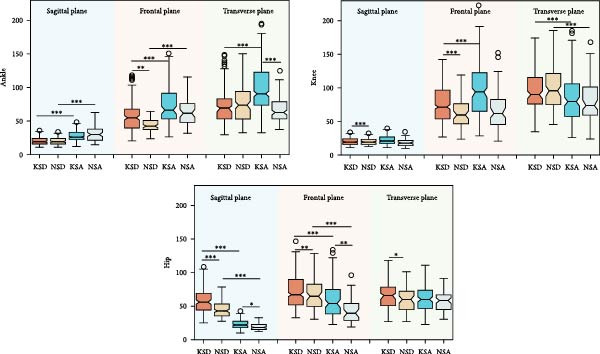
The intrasubject coefficient of variance of lower limb joint powers in sagittal, frontal, and transverse plane. ∗, ∗∗, and ∗∗∗ stand for *p* < 0.05, *p* < 0.01, and *p* < 0.001, respectively. KSD, knee osteoarthritis subjects during stair descent; NSD, normal control subjects during stair descent; KSA, knee osteoarthritis subjects during stair ascent; NSA, normal control subjects during stair ascent.

The intrasubject CVs for the foot progression angle and three‐dimensional lower limb joint angles are presented in Table 1. Table 2 shows the intrasubject CVs of GRFs in the vertical (V), AP, and ML directions, as well as the corresponding three‐dimensional lower limb joint moments. The intrasubject CVs of lower limb joint powers are summarized in Table 3. These CVs were calculated for both KOA subjects and normal controls during stair descent and ascent. The *p*‐values for the main effects of group (KOA vs. normal controls) and condition (stair descent vs. ascent), their interaction effects, and the results of post hoc analysis are reported in Tables 1–3. Post hoc analyses included between‐group comparisons (KOA vs. normal controls during both stair descent and ascent) and within‐group comparisons (stair descent vs. ascent within each group). Figures 2–4 illustrate the intrasubject CVs of joint angles, GRFs, joint moments, and joint powers, along with their corresponding significant levels for both within‐group and between‐group comparisons.

**Table 1:** The intrasubject coefficient of variance of kinematic parameters between the KOA and the normal control subjects.

Parameter	KOA		Normal		*p* value of ANOVA			*p* value of posthoc			
	Stair descent	Stair ascent	Stair descent	Stair ascent	Group	Condition	Interaction	KOA_SD-SA_	N_SD-SA_	SD_KOA-N_	SA_KOA-N_
	Mean (Std)/median [IQR]	Mean (Std)/median [IQR]	Mean (Std)/median [IQR]	Mean (Std)/median [IQR]							
FPA	14.41 [10.32, 19.26]	15.42 [12.60, 19.36]	11.41 (4.28)	13.71 (5.16)	^∗∗∗^	^∗^	0.051	0.26	^∗∗∗^	^∗∗∗^	0.12
A.F/E	12.83 [10.85, 14.98]	20.56 (5.16)	10.63 (2.81)	18.46 (6.32)	^∗∗^	^∗∗∗^	0.89	^∗∗∗^	^∗∗∗^	^∗∗∗^	^∗∗^
A.Add/Abd	18.40 [13.08, 24.14]	20.59 [14.22, 27.45]	15.11 (7.17)	17.42 [11.48, 22.85]	^∗^	^∗∗∗^	0.45	^∗∗^	^∗∗^	^∗^	0.11
A.I/Ex R.	22.87 [13.16, 44.47]	28.70 [15.53, 42.54]	15.69 [9.76, 23.78]	18.21 [11.46, 32.03]	^∗∗^	0.61	0.67	^∗^	0.064	^∗^	^∗^
K.F/E	8.01 (2.48)	7.10 [6.00, 8.43]	6.25 [5.02, 8.05]	5.69 [4.70, 6.89]	^∗∗∗^	^∗∗∗^	0.97	^∗^	0.052	^∗∗∗^	^∗∗∗^
K.Add/Abd	35.89 [22.37, 63.23]	31.29 [21.09, 63.96]	23.53 [17.90, 33.06]	21.39 [14.48, 33.22]	^∗∗^	0.081	0.49	0.16	0.81	^∗∗∗^	^∗∗∗^
K.I/Ex R.	28.92 [19.08, 51.07]	39.10 [22.24, 59.24]	21.07 [14.29, 31.43]	28.50 (14.27)	^∗∗∗^	^∗∗∗^	0.073	^∗∗∗^	^∗∗^	^∗∗^	^∗∗^
H.F/E	8.06 [6.17, 11.64]	6.19 [5.08, 8.00]	7.38 [5.65, 8.66]	4.55 [3.78, 6.07]	^∗∗∗^	^∗∗∗^	0.94	^∗∗∗^	^∗∗∗^	^∗^	^∗∗∗^
H.Add/Abd	33.36 [22.30, 46.61]	27.82 [18.59, 37.39]	29.72 (12.82)	19.07 [19.65, 39.54]	^∗∗∗^	^∗∗∗^	0.46	^∗∗∗^	^∗∗∗^	0.052	^∗∗∗^
H.I/Ex R.	37.29 [22.45, 63.87]	40.62 [20.68, 70.06]	23.23 [17.21, 38.66]	21.02 [14.90, 40.84]	^∗∗∗^	0.20	0.24	1.00	1.00	^∗∗^	^∗∗∗^
P.Tilt	15.23 [9.50, 32.42]	6.39 [4.93, 9.32]	12.77 [9.33, 28.22]	6.03 [4.20, 7.13]	0.21	^∗∗∗^	0.90	^∗∗∗^	^∗∗∗^	0.64	0.061
P.Lat.Tilt	41.33 [27.98, 61.34]	24.34 [19.28, 33.13]	30.26 [22.64, 43.56]	19.10 (5.79)	^∗∗∗^	^∗∗∗^	0.20	^∗∗∗^	^∗∗∗^	^∗∗^	^∗∗∗^
P.R.	57.21 [39.48, 95.60]	71.64 [49.16, 100.43]	66.05 [42.67, 90.59]	60.91 [39.48, 95.60]	0.65	0.056	0.44	0.096	1.00	1.00	1.00

*Note:* Descriptive statistics are presented as mean (Std) for normally distributed data and median [IQR] for nonnormally distributed data. The CV is presented as 100%. KOA_SD-SA_ and N_SD-SA_ stand for significant levels between SD and SA for KOA and normal subjects. SD_KOA-N_ and SA_KOA-N_ stand for significant levels between KOA and normal subjects during SD and SA condition. A., K., H., and P. stand for ankle, knee, hip, and pelvic segment.

Abbreviations: Add/Abd, adduction/abduction; F/E, flexion/extension; FPA, foot progression angle; I/Ex, internal/external; IQR, interquartile range; Lat, lateral; R., rotation; Std, standard deviation.

^∗^ *p* < 0.05.

^∗∗^ *p* < 0.01.

^∗∗∗^ *p* < 0.001.

**Table 2:** Intrasubject coefficient of variance (CV) of ground reaction forces (GRFs) and lower limb joint moments.

Parameter	KOA		Normal		*p* value of ANOVA			*p* value of posthoc			
	Stair descent	Stair ascent	Stair descent	Stair ascent	Group	Condition	Interaction	KOA_SD-SA_	N_SD-SA_	SD_KOA-N_	SA_KOA-N_
	Mean (Std)/median [IQR]	Mean (Std)/median [IQR]	Mean (Std)/median [IQR]	Mean (Std)/median [IQR]							
GRF_V	6.02 [4.95, 7.84]	5.09 (1.6)	5.59 [4.59, 7.45]	4.4 [3.63, 5.39]	0.068	^∗∗∗^	0.74	^∗∗∗^	^∗∗∗^	0.22	0.22
GRF_AP	18.22 [15.14, 21.80]	30.31 [24.50, 37.07]	16.67 [14.94, 20.59]	24.61 [21.65, 31.48]	^∗∗^	^∗∗∗^	^∗∗^	^∗∗∗^	^∗∗∗^	0.44	^∗∗^
GRF_ML	21.32 [17.28, 29.87]	32.25 (10.73)	24.61 [17.82, 27.41]	25.51 [20.16, 34.96]	0.073	^∗∗∗^	^∗∗^	^∗∗∗^	^∗^	0.96	^∗^
A.F/E	10.61 [8.27, 12.49]	12.81 [10.36, 16.30]	9.76 [8.51, 11.64]	12.4 [10.42, 15.19]	0.31	^∗∗∗^	0.83	^∗∗∗^	^∗∗∗^	0.55	0.55
A.Add/Abd	20.25 [13.86, 37.44]	22.43 [16.77, 37.42]	13.37 [10.36, 17.25]	24.73 [20.60, 35.61]	0.066	0.19	^∗∗∗^	0.39	^∗∗∗^	^∗∗∗^	0.31
A.I/Ex R.	32.28 [20.63, 53.74]	49.89 [34.59, 68.05]	46.16 (23.54)	28.95 [22.97, 37.99]	0.09	0.021	^∗∗∗^	^∗∗∗^	^∗∗^	0.12	^∗∗∗^
K.F/E	13.59 [10.93, 17.24]	19.06 (6.18)	12.57 [10.79, 14.72]	13.9 [11.77, 16.97]	^∗∗∗^	^∗∗∗^	^∗∗^	^∗∗∗^	^∗∗^	0.13	^∗∗∗^
K.Add/Abd	18.86 [13.26, 31.77]	31.59 [23.24, 53.14]	30.73 (21.3)	19.13 [12.02, 30.79]	^∗∗^	^∗∗∗^	^∗∗∗^	^∗∗∗^	0.23	0.61	^∗∗∗^
K.I/Ex R.	21.67 [17.10, 28.51]	26.16 (9.77)	20.54 [16.97, 26.15]	16.81 [13.37, 22.41]	^∗∗∗^	0.18	^∗∗∗^	^∗^	^∗∗^	0.8	^∗∗∗^
H.F/E	38.17 [28.82, 57.90]	18.66 [14.21, 23.22]	31.66 [23.37, 41.06]	16.95 (6.23)	^∗∗∗^	^∗∗∗^	^∗∗^	^∗∗∗^	^∗∗∗^	^∗∗^	0.059
H.Add/Abd	9.71 [8.18, 12.61]	11.04 [8.83, 15.32]	9.72 [7.35, 12.71]	7.88 [7.02, 10.34]	^∗∗∗^	^∗^	^∗∗∗^	^∗∗^	^∗^	0.37	^∗∗∗^
H.I/Ex R.	18.17 [14.84, 26.10]	15.41 [12.31, 19.71]	16.25 [12.89, 24.31]	12.06 (3.11)	^∗∗^	^∗∗∗^	0.74	^∗∗∗^	^∗∗∗^	0.15	^∗∗^

*Note:* Descriptive statistics are presented as mean (Std) for normally distributed data and median [IQR] for nonnormally distributed data. CVs are expressed as 100%. A., K., and H. stand for ankle, knee, and hip segment. V, AP, and ML stand for vertical, anterior–posterior, and medial–lateral direction. KOA_SD-SA_ and N_SD-SA_ stand for significant levels between SD and SA for KOA and normal subjects. SD_KOA-N_,and SA_KOA-N_ stand for significant levels between KOA and normal subjects during SD and SA condition.

Abbreviations: Add/Abd, adduction/abduction; F/E, flexion/extension; I/Ex, internal/external; IQR, interquartile range; R., rotation; Std, standard deviation.

^∗^ *p* < 0.05.

^∗∗^ *p* < 0.01.

^∗∗∗^ *p* < 0.001.

**Table 3:** Intrasubject coefficient of variance (CV) of lower limb joint powers.

Parameter	KOA		Normal		*p* value of ANOVA			*p* value of posthoc			
	Stair descent	Stair ascent	Stair descent	Stair ascent	Group	Condition	Interaction	KOA_SD-SA_	N_SD-SA_	SD_KOA-N_	SA_KOA-N_
	Mean (Std)/median [IQR]	Mean (Std)/median [IQR]	Mean (Std)/median [IQR]	Mean (std)/median [IQR]							
A.F/E	19.98 [16.45, 25.30]	26.25 [22.80, 33.60]	24.47 [16.77, 25.98]	30.49 (10.88)	0.21	^∗∗∗^	0.21	^∗∗∗^	^∗∗∗^	0.79	0.79
A.Add/Abd	56.26 [41.36, 73.91]	66.35 [53.21, 92.32]	42.02 [37.31, 50.36]	64.79 (20.92)	^∗∗^	^∗∗∗^	0.14	^∗∗∗^	^∗∗∗^	^∗∗^	0.35
A.I/Ex R.	69.09 [51.60, 89.17]	91.14 [76.12, 125.47]	77.53 (32.95)	66.76 [54.34, 80.78]	^∗∗∗^	^∗∗∗^	^∗∗∗^	^∗∗∗^	1.00	1.00	^∗∗∗^
K.F/E	20.63 [17.45, 23.88]	21.37 [17.70, 26.28]	18.80 [16.24, 23.39]	17.45 (5.22)	^∗∗∗^	0.12	^∗^	0.55	0.06	0.13	^∗∗∗^
K.Add/Abd	75.80 (27.29)	96.35 [64.30, 127.47]	61.00 (23.80)	68.28 [53.21, 97.24]	^∗∗∗^	^∗∗∗^	^∗∗^	^∗∗∗^	0.24	0.06	^∗∗∗^
K.I/Ex R.	87.21 [72.42, 116.21]	79.37 [56.27, 112.36]	94.71 (32.37)	73.56 [58.15, 103.68]	0.94	^∗∗∗^	0.73	^∗∗^	^∗∗^	1.00	1.00
H.F/E	56.37 [43.70, 71.33]	22.02 [17.78, 28.45]	44.42 [36.93, 57.07]	19.13 [15.51, 24.83]	^∗∗∗^	^∗∗∗^	^∗∗∗^	^∗∗∗^	^∗∗∗^	^∗∗∗^	^∗^
H.Add/Abd	67.91 [54.51, 89.69]	55.24 [39.47, 80.55]	69.78 (26.90)	39.91 [29.42, 55.33]	^∗∗^	^∗∗∗^	0.44	^∗∗∗^	^∗∗∗^	0.10	^∗∗^
H.I/Ex R.	68.15 (23.14)	59.40 [46.50, 74.10]	60.28 (18.40)	58.09 (15.74)	0.27	^∗∗^	0.32	^∗^	1.00	0.57	1.00

*Note:* Descriptive statistics are presented as mean (Std) for normally distributed data and median [IQR] for nonnormally distributed data. CVs are expressed as 100%. A., K., and H. stand for ankle, knee, and hip segment. KOA_SD-SA_ and N_SD-SA_ stand for significant levels between SD and SA for KOA and normal subjects. SD_KOA-N_ and SA_KAO-N_ stand for significant levels between KOA and normal subjects during SD and SA condition.

Abbreviations: Add/Abd, adduction/abduction; F/E, flexion/extension; I/Ex, internal/external rotation; IQR, interquartile range; R., rotation; Std, standard deviation.

^∗^ *p* < 0.05.

^∗∗^ *p* < 0.01.

^∗∗∗^ *p* < 0.001.

### 3.1. Intrasubject Variability in Kinematic Parameters

As shown in Table 1, most kinematic parameters demonstrated statistically significant main effects of group and condition (*p* < 0.05). However, no interaction effect (condition × group) was observed for any of the investigated parameters.

During stair ascent, participants in both groups exhibited greater or comparable kinematic variability in ankle and knee joints, but lower variability at the hip joint and pelvis, compared with stair descent (Figure 2 and Table 1). For both stair descent and ascent, individuals with KOA demonstrated greater kinematic variability (CVs  = 6.19%–41.33%) than healthy controls (CVs  = 4.55%–30.26%), except for pelvic rotation angle. Across all conditions and groups, sagittal‐plane joint angles showed the smallest variability (CVs  = 7.13%–32.47%), whereas coronal‐plane angles exhibited the greatest variability (CVs  = 49.39%–271.53%).

### 3.2. Intrasubject Variabilit in Kinetic Parameters

The statistical results for kinetic parameters differ from those for kinematic parameters. As shown in Table 2 and Figure 3, the main effect of group revealed significant differences in the AP GRF (*p* < 0.01) and knee joint moments (*p* < 0.001) during stair ascent. For the condition factor, significant differences were observed in GRFs, ankle flexion/extension moment (*p* < 0.001), knee joint moments (*p* < 0.001; excluding knee internal/external rotation moments), and hip joint moments, indicating distinct gait variability patterns between stair descent and ascent in both KOA and control groups. Significant interaction effects were found for GRFs and lower limb joint moments, except for the vertical GRF, ankle flexion/extension, and hip internal/external rotation moments. This suggests that KOA subjects adopted different movement strategies during stair ascent and descent compared with healthy controls.

As shown in Table 3 and Figure 4, significant main effects of condition were found for the CVs of all investigated joint powers (*p* < 0.001), except for knee flexion/extension power. Additionally, significant main effects of group were observed in most lower limb joint power parameters, with the exception of ankle flexion/extension, knee internal/external rotation, and hip internal/external rotation powers. Significant interaction effects were identified for ankle internal/external rotation, knee flexion/extension, knee adduction/abduction, and hip flexion/extension powers.

Compared with kinematic parameters, gait variability in kinetic parameters showed a generally similar pattern between the KOA and control groups during stair descent. However, more pronounced group differences were observed during stair ascent. Individuals during upstairs walking displayed greater variability in GRFs, ankle, and knee joint moments and powers, as well as hip joint moments and powers, compared with stair descent. Overall, the variability of lower limb joint moments (ankle, knee, and hip) was generally smaller than that of joint powers (Tables 2 and 3).

## 4. Discussion

The main purpose of our study is to examine the intrasubject gait variability in individuals with KOA compared to normal controls during stair descent and stair descent tasks. We analyzed kinematic and kinetic parameters based on data recorded by a 3DMC system and a customized instrumented eight‐stair staircase. We studied gait variability during stair walking from 116 individuals with KOA and 53 normal control subjects, and each subject performed at least eight gait trials for each walking condition, which is probably the largest study on this topic. Based on the results of our study, we investigated (1) the pathological gait variability and adaptations in KOA subjects during both stair ascent and descent condition; (2) the gait variability adaptation mechanisms between the stair ascent and descent condition in both KOA and normal control group. Furthermore, we also provided normative gait variability data during stair walking conditions.

### 4.1. Normative Lower Limb Kinematic and Kinetic Gait Variability Data During Stair Walking

A thorough understanding of gait variability in healthy individuals during stair walking is fundamental for identifying abnormal patterns, developing personalized treatment and rehabilitation programs, and design improved footwear and assistive devices for individuals with musculoskeletal disorders like KOA [38]. Previous research has explored gait variability in spatiotemporal parameter [17, 39–41], lower limb joint angles [17, 41], joint moments [42], and GRFs [38] during level–ground walking [21, 40], stair walking [38, 39, 41, 42], and sit–stand tasks [38] across various populations, including healthy young adults [38], senior people [40], KOA individuals [25, 26, 41], and those with neuromuscular gait disorders [21, 39]. However, to the best of our knowledge, no study has comprehensively examined three‐dimensional kinematic and kinetic gait variabilities during both stair ascent and descent in healthy subjects and individuals with KOA. Our study is the first to provide a detailed analysis of complete lower limb kinematic and kinetic variability during stair ascent and descent tasks in both healthy and KOA subjects, offering valuable normative gait data for clinical applications.

### 4.2. The Intrasubject Gait Variability

Gait variability is a critical measure in biomechanics and rehabilitation which reflects the complexity of the human neuromusculoskeletal system, influenced by physiologic factors such as neural control, muscle function, and postural stability [43, 44]. Subtle alterations in these systems impact gait variability [45–47]. Typically, healthy gait demonstrates low to moderate variability. Both excessive and minimal variability can signal potential neuromuscular issues. Increased gait variability may indicate instability or impaired motor control, heightening the risk of falls, while reduced variability might suggest a rigid or less adaptable gait pattern [43, 44].

#### 4.2.1. The Pathological Gait Variability Patterns in KOA Subjects

Our findings indicate that individuals with KOA exhibit greater gait variability in most lower limb kinematic parameters during both stair ascent and descent (Figure 2 and Table 1). This contrasts with treadmill‐walking results, where Kiss et al. [48] reported decreased variability in ankle and knee joint angles in KOA. For kinetic parameters, KOA individuals also showed generally greater variability joint moments and powers, most prominently at the knee and hip joints during stair ascent, and greater variabiltiy in ankle adduction/abduction and knee flexion/extension moments and powers during stair descent (Tables 2 and 3).

This discrepancy between stair and treadmill findings in KOA individuals likely reflects the task‐specific mechanical demands of stair negotiation, which requires larger ranges of motion and higher joint moments, especially at the knee and hip joints, when compared with treadmill walking [22]. In addition, the repetitive and externally paced nature of treadmill walking may facilitate a more controlled and conservative gait strategy in KOA, may resulting in lower gait variability [48].

#### 4.2.2. The Difference in Gait Variability Between Stair Ascent and Descent Conditions

When comparing gait variability between stair ascent and descent, parameters involving the knee and more distal segments exhibited higher CVs during ascent, whereas parameters at proximal segments (hip and pelvis), demonstrated lower CVs (Tables 1–3).

These differences reflect the task‐specific biomechanical adaptations in both KOA and healthy control subjects between stair ascent and descent. During stair ascent, the primary goal is to raise the body, which requires larger ranges of motion and higher joint moments [49], particularly at the hip and knee [49, 50], and steady control of pelvic and hip motion [44]. In contrast, the descent of the stairs emphasizes controlled energy absorption and joint stabilization to manage downward momentum [33, 49], placing greater demands on precise foot placement and segment coordination [49]. For individuals with KOA, pain, reduced strength, and limited knee mobility can amplify these task demands [28], may contributing to the greater variability we observed at the movement level.

Only a few studies have examined gait variability during stair walking. Novak and Brouwer [42] reported intrasubject variability of lower limb joint moments in the sagittal and frontal planes during stair ascent and descent in young and older adults. They found that sagittal‐plane ankle and hip joint moments variability was lower during ascent (CVs = 12.0%–24.4%) than during descent (CVs = 16.7%–38.5%) [42]. This pattern is partially inconsistent with our findings. In our study, both participants with KOA and healthy controls exhibited greater ankle flexion/extension moment variability during ascent than descent, whereas hip flexion/extension moment variability was lower during ascent than descent (Table 2). These discrepancies reflect study‐level factor such as sample size, participant characteristics, number of trials, or staircase geometry or configuration [42].

#### 4.2.3. The Different Adaptation Mechanism During Stair Walking in KOA Subjects and Normal Control Subjects

KOA individuals showed kinematic variability comparable to those of healthy controls during stair walking, as indicated by the nonsignificant interaction effect for kinematic CVs. In contrast, the kinetic results differed by task supported by the significant interaction effect: KOA and control groups exhibited different variability patterns when ascent was compared with descent (Tables 1–3). In healthy controls, CVs of kinetic parameters during ascent were similar to, or slightly lower than, those during descent (Figures 3 and 4). In KOA subjects, Kinetic CVs were higher during ascent, making it a potential marker for monitoring disease progression or recovery [51].

#### 4.2.4. The Gait Variability in Sagittal, Frontal, and Coronal Planes

Gait variability in most features of the frontal and coronal planes was greater than in the sagittal plane for both groups (Tables 1–3). Novak and Brouwer [42] reported a similar pattern, higher CVs for frontal‐plane joint moments than for sagittal plane moments in both young and old adults, which aligns with our findings (Table 2).

This plane‐specific difference likely reflects the control demands of stair walking: sagittal motions (e.g., flexion and extension) primarily support forward and upward motions and are relatively predictable [33, 49], whereas frontal‐and‐coronal‐plane control involve lateral and rotational adjustments and stabilization that are more sensitive to step‐to‐step balance requirements during complex task such as stair walking [33, 49].

The increased variability in these planes may not indicate dysfunction, but instead represent the body’s adaptive mechanism for responding to balance challenges. For populations with musculoskeletal conditions like KOA, who may have compromised neuromuscular control [48], these adjustment become even more critical. Higher variability in the frontal and coronal planes likely reflect the body’s effort to achieve dynamic stability during stair walking, where lateral and rotational control are crucial for safe navigation [44, 50].

### 4.3. Implications for KOA Assessment and Rehabilitation

For KOA assessment, our findings offer practical implications for evaluation and monitoring. First, stair walking, especially stair ascent, may be a sensitive task for detecting differences in lower‐limb joint functions, as KOA participants showed greater variability under this condition. Stair walking is more mechanically demanding than level–ground walking and can reveal subtle deficits that are less apparent during simpler tasks [42, 49]. Incorporating stair‐walking trials into clinical assessments could therefore complement standard evaluation and help gauge KOA severity [42].

Second, gait variability, especially in kinetic parameters, may serve as a candidate marker for tracking disease progression and evaluating response to rehabilitation or other treatment [48]. Monitoring stair‐walking variability can indicate how consistently joint moments and powers are produced across steps [22, 52]. Because our study did not include longitudinal data, this proposal should be tested in future prospective work.

The patterns observed during stair walking also suggest implications for KOA rehabilitation. Training that targets dynamic tasks and emphasizes coordinated control across the ankle, knee, hip, and pelvis, rather than focusing on a single joint, may help patients manage the mechanical demands of stair walking and reduce excessive loading. Even in healthy adults, such exercises may improve stair‐walking efficiency and contribute to the prevention of musculoskeletal disorders [53].

### 4.4. Limitations

This research is limited by an unequal gender distribution between groups. The imbalance male and female participation may introduce bias and affect the interpretation and generalizability of the findings, particularly with respect to sex‐specific biomechanical differences. Future research should recruit more gender‐balanced cohorts to ensure that gender‐related effects are appropriately accounted for.

## 5. Conclusion

We recruited 169 subjects, including 116 KOA subjects and 53 age‐matched healthy controls, to investigate intrasubject gait variability in kinematic and kinetic parameters during stair walking. We presented a normative dataset and investigated the pathological gait variability patterns in KOA subjects during stair walking. We found individuals with KOA showed significantly greater movement variability than controls during stair walking. This disparity was most evident during stair ascent, where KOA individuals demonstrated markedly greater variability in knee and hip joint moments and power, alongside elevated kinematic variability in both ascent and descent. These findings indicate task‐specific differences in the consistency of joint mechanics between ascent and descent in KOA. From a practical standpoint, interventions should target the entire lower limb (ankle, knee, and hip) and pelvis, with task‐specific practice for stair walking. Such programs may help patients manage the mechanical demands of stair walking.

**Nomenclature**
KOA: Knee osteoarthritis
ADL: Activities of daily living
K/L: Kellgren/Lawrence
CAST: Calibrated anatomical system technique
GRF: Ground reaction force
BW: Body weight
CV: Coefficient of variation
Std: Standard deviation
IQR: Interquartile range
V: Vertical
AP: Anterior–posterior
ML: Medial–lateral
SD: Stair descent
SA: Stair ascent
KSD: Knee osteoarthritis subjects during stair descent
KSA: Knee osteoarthritis subjects during stair ascent
NSD: Normal control subjects during stair descent
NSA: Normal control subjects during stair ascent
F/E: Flexion/extension
Add/Abd: Adduction/abduction
I/Ex R.: Internal/external rotation.

## Ethics Statement

The study was conducted in compliance with the latest version of the Declaration of Helsinki and the protocol was approved by the Ethical Committee of the Affiliated Rehabilitation Hospital at Fujian University of Traditional Chinese Medicine (Approval Numbers 2018KY‐006‐03 and 2020KS‐5‐1).

## Conflicts of Interest

The authors declare no conflicts of interest.

## Funding

This research was supported by the National Natural Science Foundation of China (Grants 12572368 and 82405535), the Natural Science Foundation of Fujian Province (Grant 2025E3005), the 2025 Ningbo University High‐Level Science and Technology Project Incubation Program (Grant GJPY2025027), the Foundation of Key Laboratory of Orthopedics and Traumatology of Traditional Chinese Medicine and Rehabilitation, Ministry of Education, Fujian University of Traditional Chinese Medicine (Grant XGS2024004), and the K.C. Wong Magna Fund in Ningbo University.

## Data Availability

The data that support the findings of this study are available from the corresponding author upon reasonable request.
